# Micro-fusion inhibition tests: quantifying antibody neutralization of virus-mediated cell–cell fusion

**DOI:** 10.1099/jgv.0.001506

**Published:** 2020-10-15

**Authors:** Nazia Thakur, Carina Conceicao, Ariel Isaacs, Stacey Human, Naphak Modhiran, Rebecca K. McLean, Miriam Pedrera, Tiong Kit Tan, Pramila Rijal, Alain Townsend, Geraldine Taylor, Paul R. Young, Daniel Watterson, Keith J. Chappell, Simon P. Graham, Dalan Bailey

**Affiliations:** ^1^​ The Pirbright Institute, Ash Road, Pirbright, Woking, GU24 0NF, UK; ^2^​ University of Queensland, Brisbane, Queensland 4071, Australia; ^3^​ MRC Human Immunology Unit, MRC Weatherall Institute of Molecular Medicine, University of Oxford, Oxford OX3 9DS, UK

**Keywords:** cell–cell fusion, enveloped virus, mFIT, neutralizing antibodies, Nipah virus, RSV, SARS-CoV, SARS-CoV-2, vaccines

## Abstract

Although enveloped viruses canonically mediate particle entry through virus–cell fusion, certain viruses can spread by cell–cell fusion, brought about by receptor engagement and triggering of membrane-bound, viral-encoded fusion proteins on the surface of cells. The formation of pathogenic syncytia or multinucleated cells is seen *in vivo*, but their contribution to viral pathogenesis is poorly understood. For the negative-strand paramyxoviruses respiratory syncytial virus (RSV) and Nipah virus (NiV), cell–cell spread is highly efficient because their oligomeric fusion protein complexes are active at neutral pH. The recently emerged severe acute respiratory syndrome coronavirus 2 (SARS-CoV-2) has also been reported to induce syncytia formation in infected cells, with the spike protein initiating cell–cell fusion. Whilst it is well established that fusion protein-specific antibodies can block particle attachment and/or entry into the cell (canonical virus neutralization), their capacity to inhibit cell–cell fusion and the consequences of this neutralization for the control of infection are not well characterized, in part because of the lack of specific tools to assay and quantify this activity. Using an adapted bimolecular fluorescence complementation assay, based on a split GFP*–Renilla* luciferase reporter, we have established a micro-fusion inhibition test (mFIT) that allows the identification and quantification of these neutralizing antibodies. This assay has been optimized for high-throughput use and its applicability has been demonstrated by screening monoclonal antibody (mAb)-mediated inhibition of RSV and NiV fusion and, separately, the development of fusion-inhibitory antibodies following NiV vaccine immunization in pigs. In light of the recent emergence of coronavirus disease 2019 (COVID-19), a similar assay was developed for SARS-CoV-2 and used to screen mAbs and convalescent patient plasma for fusion-inhibitory antibodies. Using mFITs to assess antibody responses following natural infection or vaccination is favourable, as this assay can be performed entirely at low biocontainment, without the need for live virus. In addition, the repertoire of antibodies that inhibit cell–cell fusion may be different to those that inhibit particle entry, shedding light on the mechanisms underpinning antibody-mediated neutralization of viral spread.

## Introduction

The development of neutralizing antibodies (nAbs) following infection or immunization is a central pillar of long-lasting immunity against virus infection. nAbs are principally involved in virus particle neutralization, the process by which virion entry is blocked by antibodies that inhibit receptor engagement, block uptake outright, inhibit endocytosis, cause aggregation and/or trigger complement activation [1, 2]. Accordingly, the majority of assays to identify nAbs focus on mimicking this process, involving derivations of the ubiquitous virus neutralization test (VNT) with live virus particles or pseudotyped surrogates [2–4]. Certain viruses, however, can also spread by directly inducing cell–cell fusion, resulting in multinucleated cells or syncytia [5], including severe acute respiratory syndrome coronavirus 2 (SARS-CoV-2), the recently emerged coronavirus responsible for the ongoing coronavirus disease 2019 (COVID-19) pandemic [6, 7]. Importantly, not all particle-neutralizing nAbs are capable of inhibiting cell–cell fusion and vice versa, and there are currently few robust methodologies available to specifically dissect the development and properties of fusion-inhibitory nAbs, which may be less abundant than standard nAbs, but necessary as an additional barrier to stop viral spread.

For enveloped viruses the viral glycoproteins found on the surface of virions represent the major target of nAbs. Whilst the exact mechanism of particle attachment and membrane varies, some essential principles are maintained [8]. Briefly, viral glycoproteins, often oligomeric, are embedded in the virion surface by transmembrane domains, with the majority of the protein being exterior to the virion (the ectodomain). These complexes are assembled in a pre-fusion state, maintained by intra or inter-molecular constraints, with a hydrophobic fusion loop or peptide buried within the interior of the oligomer. The capacity to initiate membrane fusion is normally primed by proteolytic cleavage of the fusogen polypeptide and triggered by ligand binding. The latter step represents the area with the greatest mechanistic heterogeneity. Viruses such as respiratory syncytial virus (RSV) and SARS-CoV-2 have a single trimeric fusogen (their fusion (F) and spike (S) proteins, respectively) that mediate both attachment and fusion, while for others, such as Nipah virus (NiV), a separate protein (glycoprotein, G) maintains the fusion (F) protein in its pre-fusion state, with triggering of F controlled by receptor binding by G. Nevertheless, the final steps are orthologous, with the fusogens undergoing large structural rearrangements to embed the fusion peptide in the host membrane, leading to the so-called post-fusion state. This process ultimately leads to the juxtaposition of viral and host membranes, the formation of a fusion pore and viral genome entry. From a nAb perspective, the development of antibodies against the pre-fusion state of the viral fusogen is considered to be favourable, as this is more likely to inhibit particle entry.

**Fig. 1.**
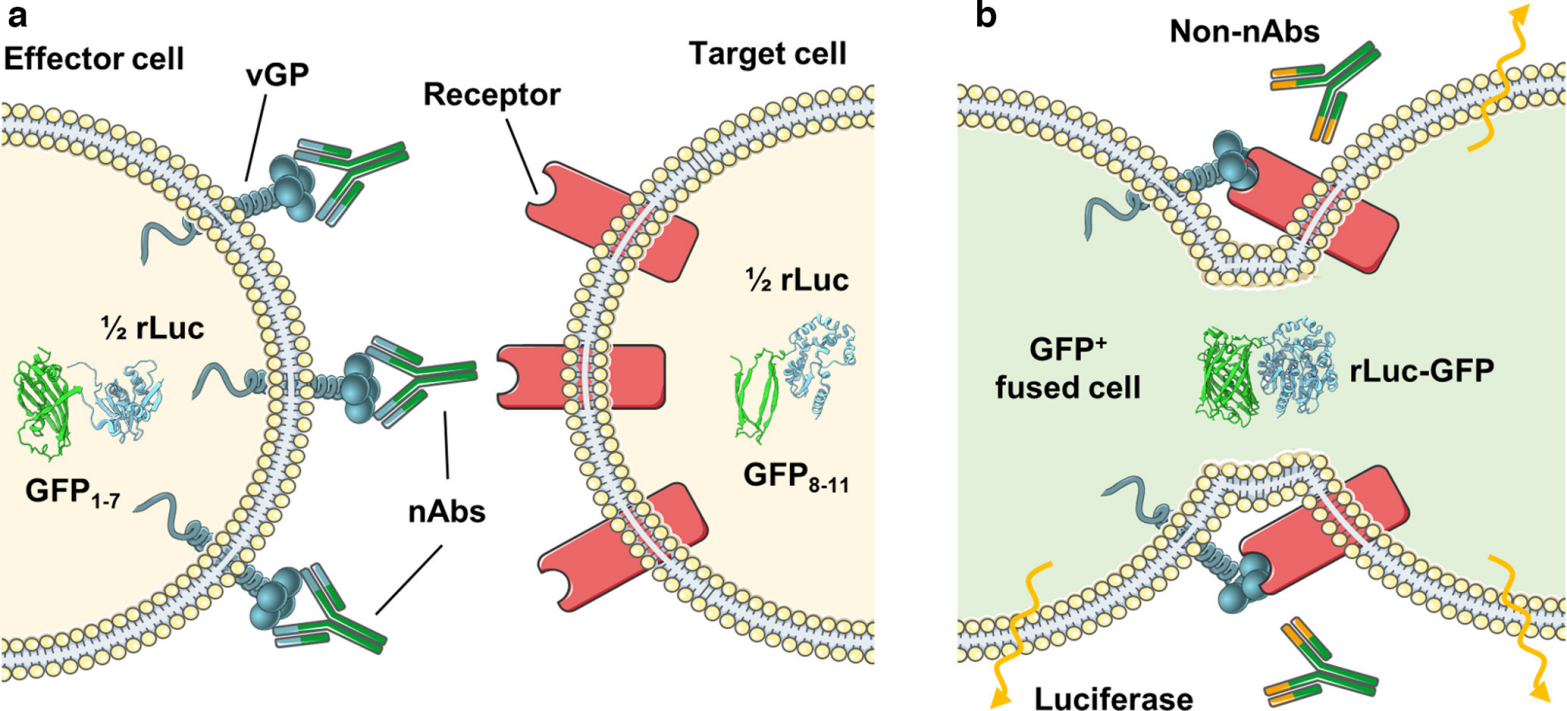
The micro-fusion inhibition test (mFIT). Samples containing antibodies are incubated with effector cells (HEK293T Lenti rLuc-GFP 1–7) expressing the viral glycoprotein (vGP) of interest. The antibody–effector cell mix is then co-cultured with target cells (HEK293T Lenti rLuc-GFP 8–11) expressing the corresponding vGP’s cellular receptor and incubated for 18–24 h. In (a) the presence of fusion-inhibitory neutralizing antibodies (nAbs) prevents the reconstitution of the rLuc-GFP reporter in fused cells, while in (b) the absence of specific neutralizing antibodies (non-nAbs), allows vGP-mediated cell–cell fusion to occur. Subsequent mixing of the target and effector cell cytoplasm leads to reconstitution of the split reporter and increased GFP and luciferase signals. This figure was generated using modified images from SMART Servier Medical Art By Servier, used under CC BY 3.0, https://smart.servier.com/, accessed June 2020.

In previous studies, we and others have developed assays to reliably quantify cell–cell fusion induced by viruses [9–14]. Indeed, these systems have been successfully applied to examine virus host range [15], protein–protein interactions within the attachment complex [16], the mechanism of attachment and entry [11, 17], and other biologically relevant questions. Seeking to adapt these systems to examine fusion-inhibitory antibodies has proven technically challenging, as many of these assays are not easily adaptable to protocols that require titration of sera or purified antibodies. However, the cell–cell fusion assay we have developed for paramyxoviruses [16, 18] has proven to be applicable to a 96-well plate format with robust repeatability [13, 15] and also rapidly adaptable, for example to SARS-CoV-2 [19]. This system is based on the use of a split green fluorescent protein*–Renilla* luciferase reporter (rLuc-GFP) to monitor cytoplasmic mixing after cell–cell fusion [20]. Briefly, ‘effector’ cells expressing rLuc-GFP 1–7 (beta strands 1–7 of GFP) and individual viral glycoproteins (vGPs) are co-cultured with ‘target’ cells expressing the rLuc-GFP 8–11 (beta strands 8–11 of GFP) component and the viral receptor. When receptor engagement triggers cell–cell fusion, the cytoplasm of target and effector cells mix and the corresponding elements of the rLuc-GFP reporter reconstitute, becoming biologically active and quantifiable. In the micro-fusion inhibition test (mFIT) described herein, we have modified this assay to allow the incubation of effector cells with sera or monoclonal antibodies (mAbs), to quantify their fusion-inhibition phenotype (Fig. 1). Whilst related assays have been described in the past [21], we believe that the mFIT provides a simple, high-throughput and tractable assay that is easily adapted to different viruses.

Our initial focus in this study was the development of mFITs for three medically and agriculturally important paramyxoviruses; human and bovine respiratory syncytial virus (h/b RSV; closely related orthopneumoviruses) and NiV (a henipavirus). hRSV is a significant cause of respiratory disease, morbidity and mortality in children and the elderly, whilst bRSV causes respiratory disease resulting in large losses to the global dairy and cattle industry. NiV is an emerging zoonotic pathogen, currently restricted to South and South-East Asia, which can be transmitted from its natural reservoir – fruit bats – to humans, causing a highly fatal respiratory and neurological disease. In the first outbreak of NiV in Malaysia in 1999 the virus also infected pigs, which served as an amplification reservoir allowing spread to farm workers [22]. For both NiV and hRSV there are no licensed vaccines for use in humans; with the only licensed therapeutic being a monoclonal antibody against the hRSV F protein [23, 24]. Following the emergence of SARS-CoV-2 in late 2019, and its pandemic spread from early 2020, we also developed an equivalent assay for this coronavirus, alongside SARS-CoV, to facilitate the examination and characterization of fusion-inhibitory antibodies against this virus. Importantly, for all the viruses described here there is established evidence (or emerging for SARS-CoV-2 [25–27]) that viral syncytia form in the tissues of infected individuals or animal models of disease [3, 28–33], highlighting the importance of this route of spread. We hope that the development of mFIT assays for these viruses will enable a broader understanding of the mechanisms of virus neutralization and the immune response to fusogenic viruses, and in doing so facilitate the development of novel vaccines and therapeutics.

## Results

### Optimization of cell–cell fusion assays

Previous iterations of the cell–cell fusion assay have relied on transient expression (via transfection) of both the vGPs and the rLuc-GFP reporter [9, 13, 34]. To simplify our approach, we clonally selected stable cell lines that express the rLuc-GFP reporter elements (Fig. S1, available in the online version of this article) and compared their activity to standard transient transfection (Fig. S2), confirming their suitability for cell–cell fusion experiments. Within our laboratory we have developed cell–cell fusion assays for a number of vGPs [13, 15, 18, 19]; however, the conditions required for fusion are rarely maintained between these proteins. For this study, the mass and ratio of DNA transfected, the length of co-culture, as well as the duration and temperature of nAb incubation, were all conditions that required optimization for hRSV, bRSV, NiV, severe acute respiratory syndrome coronavirus (SARS-CoV) and SARS-CoV-2 mFITs (Figs S3 and S4).

### RSV mFITs to examine mAb neutralization of fusion

**Fig. 2.**
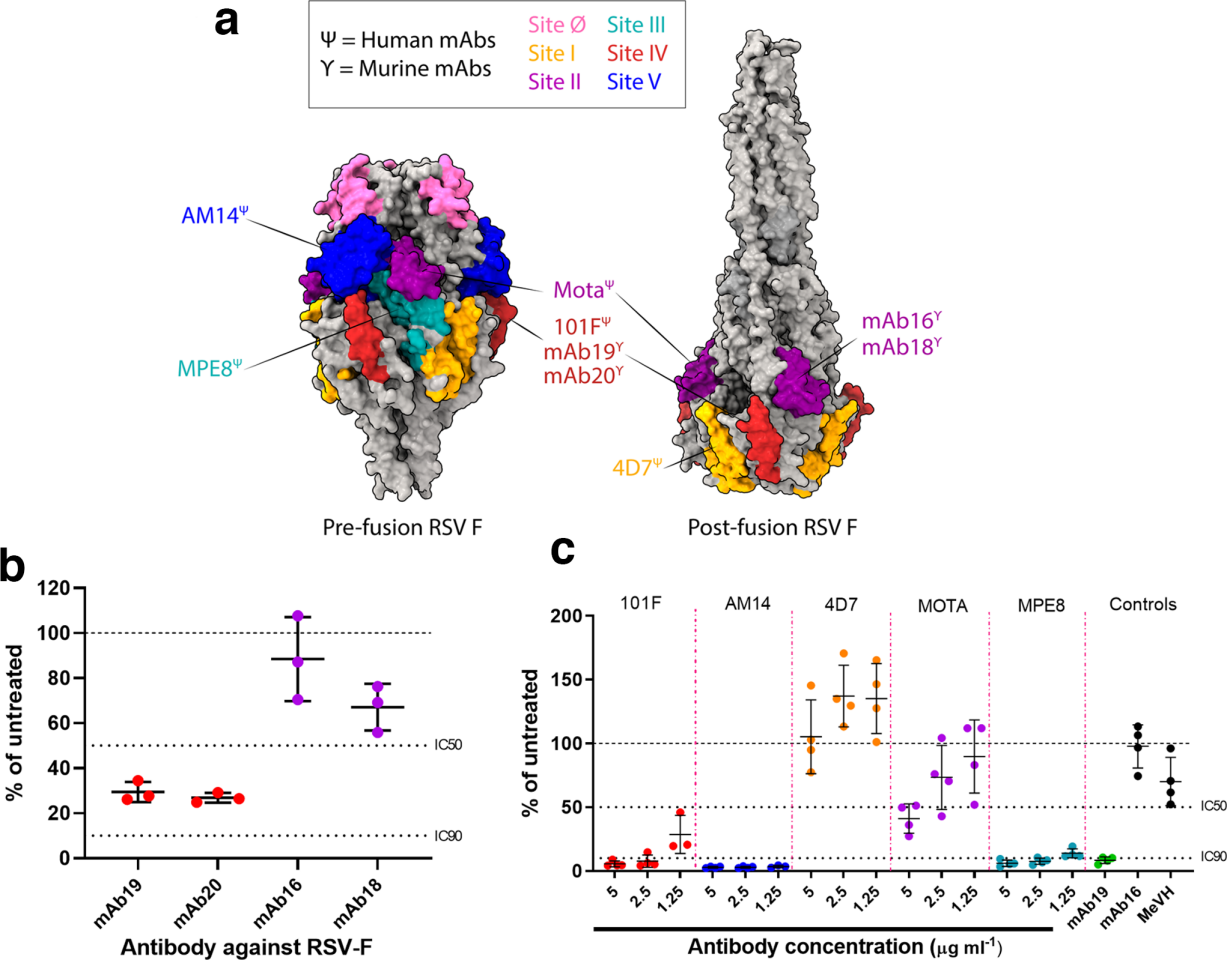
Examining the neutralization of cell–cell fusion by monoclonal antibodies in human RSV mFITs. (a) Molecular surface representation of RSV F trimer in the pre-fusion (left; PDB 4MMV) and post-fusion (right; PDB 3RRR) forms with antigenic sites coloured as follows: site ø, pink; site I, orange; site II, purple; site III, turquoise; site IV, red; site V, blue. RSV F-specific mAbs are annotated and coloured according to the corresponding antigenic binding site. Ψ represents human mAbs, while γ represents murine mAbs. Molecular graphics and analyses were performed in UCSF’s ChimeraX program. (b) Murine mAbs (1 : 160 working dilution) and (c) human mAbs (5, 2.5 and 1.25 µg ml^−1^) were tested in hRSV-F mFITs. mAb 19, positive control; mAb16, specific negative control; MeVH, non-specific negative control (Table S1). Data are expressed as a percentage of the average luciferase readings seen in no-sera/negative controls with 50 or 90 % inhibition (IC_50_ and IC_90_) lines indicated. Error bars represent mean±sd.

Using the established hRSV mFIT we began by characterizing the fusion-inhibition properties of a number of recognized human F-specific mAbs, including 101F, AM14, 4D7, Motavizumab (MOTA) and MPE8 (Fig. 2; additional mAb details provided in Table S1) [35–39]. We also included four RSV F-specific murine antibodies, which we have previously shown to either inhibit (mAb 19 and 20) or not inhibit (mAb 16 and 18) RSV fusion, albeit in semi-quantitative assays [40]. The RSV-F protein, a functional trimer, has a number of well characterized surface epitopes, which are differentially exposed on the pre- and post-fusion variants of this complex [41] (Fig. 2a), with our panel of mAbs covering a diversity of these epitopes (Fig. 2a).

Consistent with previous findings [40], murine RSV-specific mAbs 19 and 20 inhibited hRSV–F fusion, while mAbs 16 and 18 did not (Fig. 2b). Unsurprisingly, the site I-binding antibody 4D7 showed no capacity for inhibiting hRSV-F-mediated fusion in our mFIT, correlating with this mAb binding only the post-fusion variant of this epitope. In contrast, antibody binding sites II and IV, which are able to access their epitopes on both the pre- and post-fusion forms of RSV-F, showed much greater capacity for inhibition in our mFIT. 101F (site IV) showed robust inhibition of hRSV–F fusion even at 1.25 µg ml^−1^, while inhibition by MOTA (site II) gradually titred out between 5 ^1^ and 1.25 µg ml^−1^. AM14 (site V) and MPE8 (site III), which bind to the pre-fusion form of RSV-F, were also able to potently inhibit RSV fusion even at the lowest concentration assessed, 1.25 µg ml^−1^ (Fig. 2c). As an additional control we also included monoclonal antibodies to measles virus (MeV) H, which had little appreciable effect on RSV-mediated fusion. Of note, MOTA is a derivative of the licensed RSV mAb Palivizumab and MPE8 is known to cross-compete with Palivizumab [41]. The binding epitope of MPE8 is well conserved between all orthopneumoviruses – hRSV, bRSV and pneumonia virus of mice (PVM) [41] – so we carried out a side-by-side comparison of these antibodies in a bRSV-F mFIT, which demonstrated a similar trend of inhibition with both the murine and human mAbs (Fig. S5). Our findings highlight the antigenic similarity of these viruses and the likely conservation of epitopes between related F proteins, which are roughly 80 % identical at the amino acid level [42]. More broadly, we demonstrate that the mFIT can be rapidly used to screen the fusion-inhibitory properties of a panel of vGP-specific mAbs.

### NiV mFITs to examine mAb neutralization of fusion and immune responses during vaccination

**Fig. 3.**
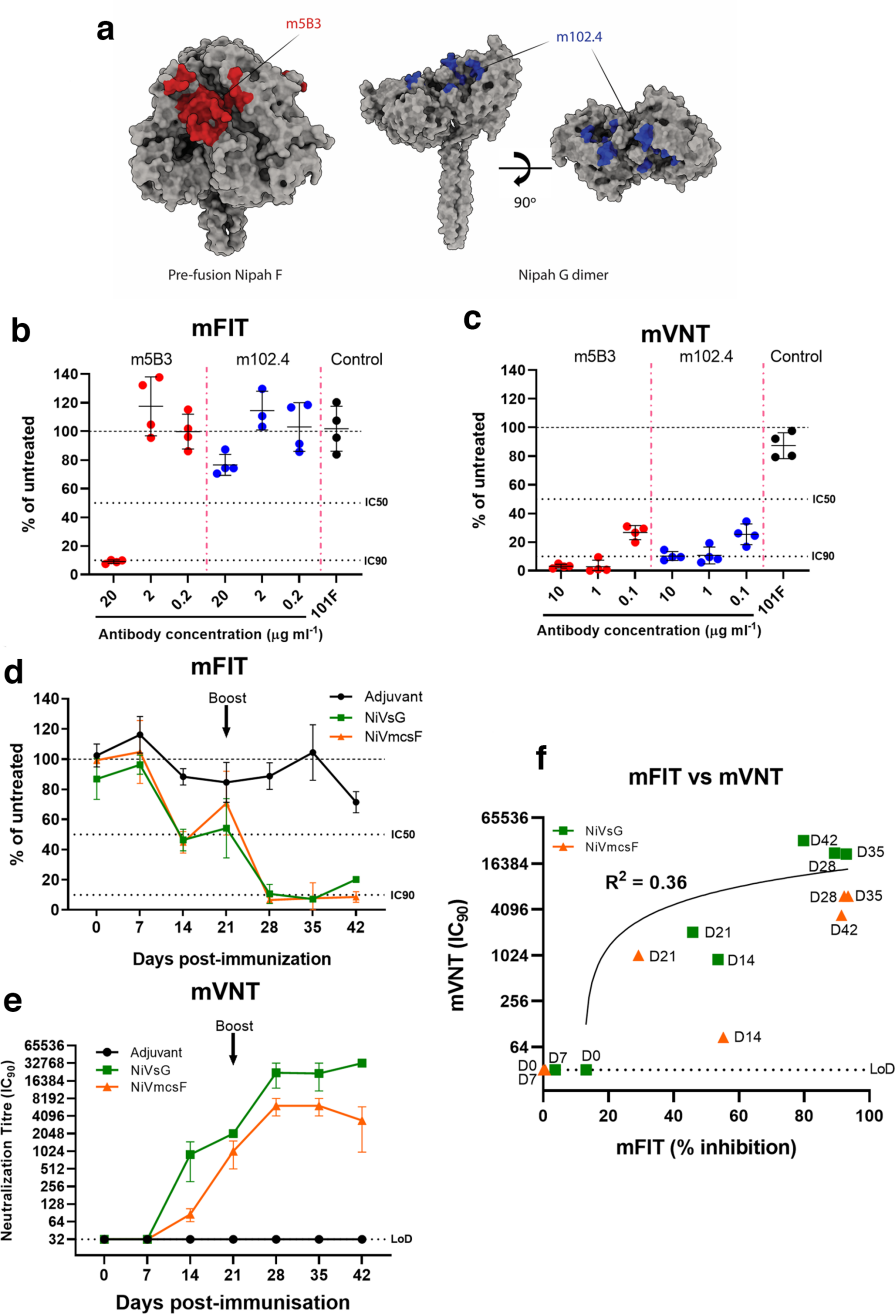
Using NiV mFITs to characterize neutralization of cell–cell fusion by monoclonal antibodies and sera from NiV vaccinated pigs. (a) Molecular surface representation of NiV-F pre-fusion trimer (left, PDB 5EVM) and NiV G dimer (right, PDB 2VWD). The stalk domain of G (residues 62–117) is modelled after parainfluenza virus 5 stalk (PDB 4JF7). The 5B3 epitope on Nipah F is coloured red, and the m102.4 epitope on Nipah G is coloured blue. Molecular graphics and analyses performed with UCSF ChimeraX. Antibodies against NiV-F- and NiV-G-specific mAbs were tested in a NiV-FG (b) mFIT (20, 2 and 0.2 µg ml^−1^) and (c) mVNT using NiV viral pseudotypes (10, 1 and 0.1 µg ml^−1^). A negative control, RSV F mAb (101F), was also included. (d) Sera from individual representative NiV mcsF- or NiV sG-vaccinated pigs were tested longitudinally in a NiV-FG mFIT (1 : 5 working dilution) and by (e) mVNT using NiV viral pseudotypes. (f) An *xy* scatter plot illustrating the correlation between mFIT results (% reduction) and mVNTs (IC_90_) from the immunogenicity study performed in pigs (Table S2). A linear line of regression is shown together with the calculated *R* ^2^ value. Data are expressed as a percentage of the average luciferase readings seen in no-sera/negative controls. Error bars represent mean±sd with 50 or 90 % inhibition (IC_50_ and IC_90_) and limit of detection (LoD) lines are indicated.

To broaden our study to examine neutralization of other fusogenic viruses, we performed NiV mFITs with two henipavirus-specific mAbs. Firstly, the human monoclonal m102.4, which specifically interacts with the receptor-binding domain (RBD) of the NiV attachment protein (G) and has strong cross-reactivity between NiV and the closely related Hendra virus (HeV) [43] (Fig. 3a). m102.4 was shown to be protective in several animal studies and has also been administered to individuals at high risk of exposure to HeV [3, 44–46]. The second monoclonal (humanized m5B3) recognizes the pre-fusion forms of NiV and HeV-F proteins and has been shown to inhibit membrane fusion by holding F in its pre-fusion form [47] (Fig. 3a). We found that the F-binding m5B3 was able to robustly inhibit fusion at 20 µg ml^−1^, which titred out at 2 µg ml^−1^. Interestingly, the G-specific m102.4 was also able to inhibit NiV fusion at 20 µg ml^−1^, albeit at reduced levels (Fig. 3b). In contrast to our mFIT results, both m102.4 and m5B3 were able to inhibit NiV particle entry at 10 µg ml^−1^ in micro virus neutralization tests (mVNTs) using pseudotyped NiV, with this inhibition beginning to titre out at 0.1 µg ml^−1^ (Fig. 3c). Of note, we included the RSV F mAb 101F as a negative control, with this mAb showing no significant inhibition of NiV-mediated fusion in either assay. These differences highlight how mFITs and VNTs can be used in tandem to probe the neutralizing properties of antibodies against vGPs.

Beyond mAb characterization, we also assessed the applicability of the mFIT in vaccine immunogenicity studies. As part of an ongoing study, we are assessing the immunogenicity and efficacy of candidate NiV vaccines in pigs. Two of the vaccine candidates undergoing analysis are recombinant, secreted variants of the NiV glycoproteins; NiV F (mcsF) (Young P.R. *et al*., in press) and NiV G (sG) [48]. These two proteins are the major targets for particle-neutralizing antibodies, which correlates well with protection from disease in *in vivo* models [3]. To date, however, little is known about the cell–cell fusion-inhibitory phenotype of these nAbs. For NiV vaccines the development of fusion-inhibitory responses may correlate significantly with immunity, since syncytia cell formation has consistently been observed in the infected tissues of experimentally infected animals [29, 49–52]. Using a homologous prime (day 0) and boost (day 21) regime, our NiV-F and NiV-G vaccine candidates were inoculated into pigs and blood samples were taken every week until 42 days post-vaccination. Sera from some of these vaccinated animals, as well as from an adjuvant-only control group, were examined for cell–cell fusion-inhibitory Abs. mFIT results from single representative animals from each group demonstrate that a fusion-inhibitory response is generated by both NiV-F and NiV-G vaccines pre-boost and that this response is significantly boosted following the second inoculation of the recombinant protein on day 21 (Fig. 3d). The development of fusion-inhibitory Abs is specific to the vaccine groups, as the adjuvant-only immunized animal did not develop equivalent responses (Fig. 3d). We were also able to show a positive correlation (Pearson *R*=0.66, *R* ^2^=0.43) between fusion inhibition and particle neutralization titres using NiV pseudotypes in mVNTs (Fig. 3e, f, Table S2). In summary, the mFITs carried out using NiV-specific mAbs and sera from vaccinated animals highlight the broad applicability of this assay and the presence of nAbs that inhibit particle fusion, cell–cell fusion, or both.

### SARS-CoV-2 and SARS-CoV mFITs to examine mAb neutralization of fusion

**Fig. 4.**
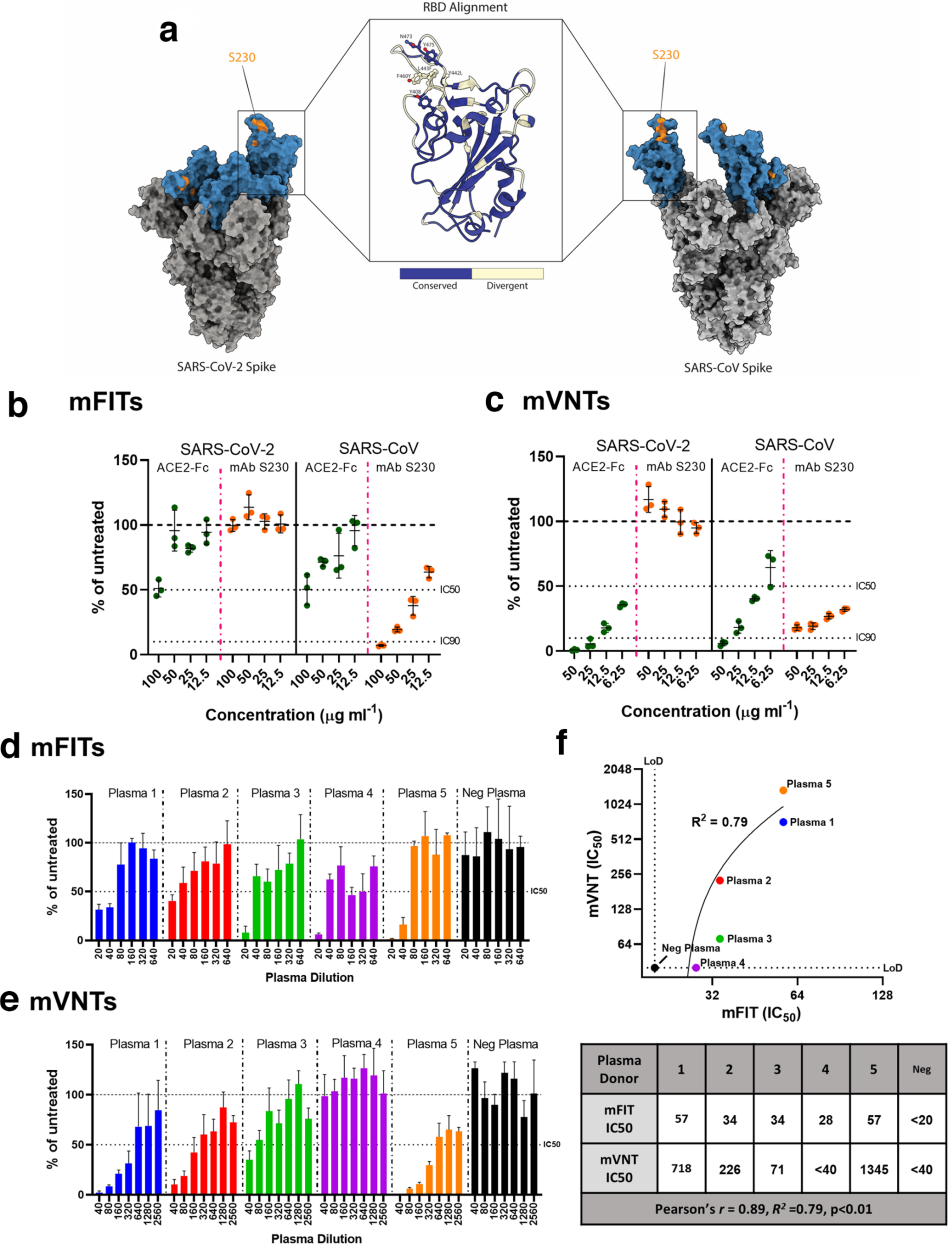
Examining neutralization of fusion by monoclonal antibodies and convalescent patient plasma in SARS-CoV-2 mFITs. (a) Molecular surface representation of SARS-CoV-2 and SARS-CoV spike trimers, with the S230 binding epitope highlighted in orange, and an RBD alignment between the two spikes shown. Molecular graphics and analyses performed with UCSF ChimeraX. A soluble ACE2-Fc and a mAb targeting SARS-CoV, S230, were tested in (b) SARS-CoV-2 and SARS-CoV mFITs (100, 50, 25 and 12.5 µg ml^−1^) and (c) mVNT using SARS-CoV-2 and SARS-CoV viral pseudotypes (50, 25, 12.5 and 6.25 µg ml^−1^). Convalescent human plasma from COVID-19 recovered patients and a negative plasma pool from healthy donors were tested in a (d) SARS-CoV-2 mFIT (1 : 20 final dilution) and by (e) mVNT using SARS-CoV-2 viral pseudotypes. (f) An *xy* scatter plot illustrating the correlation between IC_50_ results from (d) and (e). A linear line of regression is shown together with the calculated *R* ^2^ value and the Pearson’s correlation factor, *R*, all calculated from the tabulated data (under). Data are expressed as a percentage of the average luciferase readings seen in no-sera/negative controls with 50 or 90 % inhibition (IC_50_ and IC_90_) and limit of detection (LoD) lines are indicated. Error bars represent mean±sd.

Both SARS-CoV and SARS-CoV-2 S proteins, functional trimers (Fig. 4a), were rapidly adaptable to our cell–cell fusion system. We first examined whether a soluble version of the ACE2 receptor fused to the human IgG1 Fc (hinge-CH2-CH3) (ACE2-Fc) was able to inhibit S-mediated fusion. ACE2-Fc was able to inhibit approximately 50 % of SARS-CoV and SARS-CoV-2 fusion when used at 100 µg ml^−1^ with corresponding SARS-CoV and SARS-CoV-2 pseudoparticles being neutralized at lower concentrations in mVNTs (Fig. 4b, c). As proof of principle, we also examined whether the SARS-CoV-specific mAb S230 could inhibit SARS-CoV fusion in an mFIT. S230 binds the SARS-CoV S receptor-binding domain RBD; however, this epitope is not completely conserved in SARS-CoV-2 S (Fig. 4a) [53, 54]. While S230 was able to potently inhibit SARS-CoV cell–cell fusion and pseudoparticle entry, this was not the case for SARS-CoV-2 (Fig. 4b, c). This finding is congruent with previous studies investigating S230 escape mutants, demonstrating that residue L443 in SARS-CoV S is central and key for S230 binding [53]. An alignment of SARS-CoV and SARS-CoV 2 RBDs revealed this residue to be divergent between the two coronaviruses, resulting in reduced S230 binding and functionality (Fig. 4a). Nevertheless, these results highlight the potential importance of the mFIT for screening SARS-CoV-2 therapeutic candidates mAbs.

There is also an urgent need to develop a better understanding of antibody responses to SARS-CoV-2. For example, very little is known about whether antibodies can block S-mediated cell–cell fusion. With this in mind, we performed mFITs on plasma from convalescent donors who have recovered from COVID-19, alongside a negative plasma pool (from healthy donors collected prior to 2019). We found that all five donors were able to inhibit SARS-CoV-2 cell–cell fusion by >50 % when their plasma was used at a 1 : 20 dilution, although this dropped to 2/5 at the next dilution (1 : 40). As expected, the negative plasma was unable to inhibit fusion (Fig. 4d). We also demonstrated a good positive correlation (Pearson *R*=0.89, *R* ^2^=0.79) between mFIT IC_50_ values calculated for each of these donors and the corresponding IC_50_ values from mVNTs (Fig. 4e, f), allowing us to conclude that SARS-CoV-2 infection can lead to the development of cell–cell fusion-inhibitory nAbs.

## Discussion

Herein we have demonstrated the broad applicability of the viral mFIT, examining neutralization of cell–cell fusion by monoclonal antibodies and recombinant proteins as well as sera from vaccinated animals and plasma from naturally infected individuals. This assay allows the de facto calculation of fusion-inhibitory IC_50_ and IC_90_, which we demonstrated correlated well with more classical approaches such as mVNTs. While virus particle neutralization and antibody-mediated inhibition of viral-induced cell–cell fusion are likely to functionally overlap, based on the mutual recognition of epitopes on the surface of viral glycoproteins and the subsequent blocking of their related mechanisms of action, there may be distinct nAb clones that neutralize particles but cannot block fusion or vice versa. The combined use of mFITs and VNTs could prove useful in delineating these differences. This assay builds on existing techniques for the identification of antibody-mediated inhibition of cell–cell fusion, including classical observation of syncytia formation by microscopy, computational analysis of images to quantify syncytia formation, HIV-based variations of the dual-reporter assay [12] and assays based on the quantification of cellular electrical impedance [55]. Clearly, from our data, the mFIT can be applied to different viruses, dependent only on the availability of a cloned viral glycoprotein capable of inducing fusion. On this, we have also demonstrated that this assay is rapidly adaptable, in our case for SARS-CoV-2. The detection of fusion-inhibitory responses in human plasma from COVID-19 convalescent individuals highlights the clinical relevance and potential uses of this assay, especially important when considering the emerging evidence that SARS-CoV-2 causes syncytia formation in the lungs of infected individuals [25, 27]. We propose that the mFIT could be used in the assessment of COVID-19 vaccine immunogenicity and efficacy trials as well as in the assessment of candidate therapeutic mAbs, and hypothesize that the development of a highly fusion-inhibitory antibody response may correlate well with protection from infection. Separately, the mFITs for bRSV, hRSV and NiV have allowed us to functionally probe the fusion-inhibitory activity of well-characterized RSV F and NiV mAbs, including those approved for use or in clinical trial. The sensitivity of RSV fusion to the mAbs tested supports their therapeutic use, since this virus is known to spread via fusion in the lungs of infected individuals [56]. This inhibition also correlated well with our understanding of the RSV F protein and which epitopes are available for Ab-binding on the pre- and post- fusion variants of the protein. In related immunogenicity studies we were also able to monitor the emergence of a fusion-inhibitory nAb response following NiV vaccination in pigs. When interpreting the inhibitory capacity of antibodies used in this assay, it is important to consider the potential mechanisms of action of these antibodies. Inhibition may not necessarily correlate to direct inhibition of the glycoprotein fusion machinery, but instead inhibition of the steps leading up to fusion. We therefore propose three mechanisms of action: (1) antibodies block attachment, preventing receptor engagement and downstream activation of fusion, e.g. m102.4, which interacts with the RBD of NiV-G, not NiV-F; (2) antibodies bind sites distal to the RBD, blocking the activation of F by preventing conformational changes from a pre- to post- fusion state; (3) antibodies bind on or near the fusion peptide, directly inhibiting this domain being embedded in the host cell membrane. To date, our system has been optimized for viral glycoproteins that are physiologically active at neutral pH; however, we believe adaptation for glycoproteins that require a lower pH to trigger fusion is feasible. Indeed, we have already demonstrated that lowering the pH in VSV-G-transfected cells leads to cell–cell fusion (data not shown) and we are currently performing similar experiments with influenza HA, using a transient pH activation of effector cells to activate the fusogen prior to co-culture.

In general, the amount of mAb or sera required to elicit neutralization of fusion is roughly twice that required for mVNTs. This reflects both the prevalence of fusion-inhibitory nAbs in sera and the level of viral glycoproteins available to induce fusion on the surface of transfected cells. There is likely to be more vGP present on the surface of an effector cell in our fusion assay compared with that on the surface of a pseudoparticle in a mVNT. Indeed, the constructs used to drive vGP expression are codon-optimized for human cells, which probably increases the amount of antibody required to inhibit fusion. This aspect of our assay in particular requires careful optimization as a balance needs to be struck between fusion and establishing a window for antibody-mediated inhibition of this process. Of note, RSV codon optimization is fundamental to the success of the mFIT, as expression of non-codon-optimized, RSV wild-type F ORF does not result in detectable levels of fusion (data not shown). There are some notable exceptions to this general trend, however, specifically those antibodies or proteins that target receptor interactions (ACE2 and m102.4). In this case neutralization in the mVNTs was far more efficient than in the mFITs, likely because cells are already in close proximity in the cell–cell fusion assay, with vGP abundance and Ab accessibility presumably constraining neutralization. Although we did not identify any instance where mFIT neutralization was more potent than mVNT, the identification and characterization of such a protein, antibody or antiviral in the future would be of broad interest. There are other technical aspects to this assay that also require consideration. One of the key conclusions to be drawn from our optimization is that too much transfected DNA is inhibitory, perhaps due to vGP overexpression and cytotoxicity. In addition, the kinetics of fusion can vary. For example, NiV effector cells are markedly more fusogenic 2 days post-transfection, while for RSV 1 day leads to superior fusion (Fig. S3). In addition, the varying fusogenicity of vGPs may impact on antibody-mediated inhibition of cell–cell fusion. To inhibit NiV fusion, for example, 20 µg ml^−1^ of inhibitory antibody is required, whereas for RSV 5 µg ml^−1^ or less was needed. However, NiV is demonstrably more fusogenic than RSV in our assay [higher luciferase signal and greater number and size of GFP-positive syncytia (Fig. S3)]; as such, side-by-side comparison of different viruses and the antibody concentrations required for inhibition of fusion might not be appropriate.

To summarize, we propose the mFIT as a complementary assay to classical and pseudotype-based VNTs, allowing additional aspects of the nAb response to infection and vaccination to be probed. This assay may prove broadly applicable for characterizing nAb responses to vaccines or therapeutics, allowing its development as a diagnostic tool for related viruses. This depth of understanding promises to greatly inform immediate requirements for efficacious SARS-CoV-2 vaccines and mAb therapeutics, and to improve our understanding of the dominant epitopes for nAbs on SARS-CoV-2 S.

## Methods

### Cell lines

Human embryonic kidney (HEK) 293T and baby hamster kidney (BHK)-21 cells (Central Services Unit, The Pirbright Institute, UK) were used for pseudoparticle generation and mVNTs and were maintained using DMEM-10%: Dulbecco’s modified Eagle’s medium (DMEM; Sigma-Aldrich) supplemented with 10 % foetal bovine serum (FBS; Life Science Production), 1 % sodium pyruvate, NaP solution (Sigma-Aldrich) and 1 % penicillin/streptomycin (Pen-Strep; 10 000 U ml^−1^; Life Technologies Ltd). HEK293T cells (Cell Servicing Unit, The Pirbright Institute, UK) stably expressing Lenti-rLuc-GFP 1–7, or separately, Lenti-rLuc-GFP 8–11 were used for all fusion assays and mFITs and were maintained using PRF-DMEM-10%: phenol red-free DMEM (Sigma-Aldrich) supplemented with 10 % FBS (Life Science Production), 1 % NaP (Sigma-Aldrich), 1 % Pen-Strep (10 000 U ml^−1^; Life Technologies Ltd) and 1 % l-glutamine 200 mM (Sigma-Aldrich).

### Stable cell line generation

HEK293T cells were transduced with lentiviruses expressing halves of a split *Renilla*–GFP luciferase (rLuc-GFP) reporter, rLuc-GFP 1–7 or rLuc-GFP 8–11 [20]. Lentiviral plasmids expressing these constructs were generated using plasmids provided by Zene Matsuda, University of Tokyo as a template along with primers rLuc-GFP 1–7 (forward: AATTACTAGTGCCACCATGgcttccaaggtgtacgacccc, reverse: AATTACGCGTTTATcacttgtcggcggtgatgta) or rLuc-GFP 8–11 (forward: AATTACTAGTGCCACCATGcagaagaacggcatcaaggcc, reverse: AATTACGCGTTTAttactgctcgttcttcagcac), generating amplicons via PCR using KOD DNA polymerase (Novagen) as follows: 95 °C for 2 min, followed by 30 cycles of 95 °C for 20 s, 58 °C for 10 s and 70 °C for 15 s, and yielding PCR products of ~951 and ~711 bp, respectively (Fig. S1a). These products were then cloned separately into a lentiviral vector encoding a puromycin selection marker (pdlNotIMC S’R’Pk – Lenti wild-type empty) using *SpeI* and *MluI* restriction enzymes (New England Biolabs). Lentiviral vectors encoding the protein of interest (1 µg) were transfected into HEK293T cells along with two helper plasmids encoding the VSV-G (0.5 µg) and Gag/Pro/Pol, Tat and Rev proteins of HIV-1 (0.5 µg) in the pcDNA3.1 backbone using the *Trans*It-X2 Dynamic Delivery System (Geneflow) as per the manufacturer’s recommended protocol. After 48 h, cell supernatants containing the lentivirus were collected, centrifuged at 1800 ***g*** for 10 min and filtered through a 0.45 µm syringe filter. One millilitre of the lentivirus-containing medium was then transduced onto HEK293T cells with 5 µg ml^−1^ polybrene (Sigma-Aldrich) for 2 h, after which cells were supplemented with 2 ml DMEM-10 % and incubated for a further 48 h. Cells were then expanded and clonally selected under 1 µg ml^−1^ puromycin (Gibco) selection.

### Plasmids

rLuc-GFP 1–7 and rLuc-GFP 8–11 plasmids (available upon request under MTA from Zene Matsuda, University of Tokyo [20]) were used to test the stable cell lines that were generated. RSV-specific fusion assay optimization and mFITs in combination with mAbs were carried out using bRSV-F (Snook strain, Y17970.1) or a codon-optimized hRSV-F (A2 strain, EF566942.1). pGEN2.1 plasmids expressing codon-optimized NiV-F and NiV-G ORFs (Malaysia strain, AY816748.1 and AY816745.1, respectively), tagged at their C termini with haemagglutinin (HA) and myc, respectively, were used for initial optimization of fusion assays and subsequently used in an equivalent ratio for mFIT and mVNT assays in combination with animal sera or mAbs. pcDNA3.1 plasmids expressing codon-optimized SARS-CoV-2 spike, S (Wuhan strain QHR63290.2) or SARS-CoV S (ShanghaiQXC2 strain, AAR86775.1), tagged at their C termini with FLAG, and human angiotensin-converting enzyme 2 (hACE2, Addgene), were used to optimize fusion assays and subsequently used in mFITs and mVNTs with mAbs or human plasma.

### Sera and monoclonal antibodies

Weekly sera samples were obtained from 10-week-old, female, Large White–Landrace–Hampshire cross-bred pigs immunized with recombinant vaccine candidates against the NiV glycoproteins (a molecular clamp stabilized NiV-F; NiV mcsF, or a secreted NiV-G; NiV sG), or an adjuvant-only control, as part of a yet unpublished study that followed a homologous prime–boost regime (immunization with 100 µg protein by intramuscular injection at 0 and 21 days). Convalescent plasma from donors that recovered from COVID-19 (cat no. 20/118, plasma 1–4; cat no 20/130, plasma 5) and a negative plasma pool from healthy donors collected before 2019 (cat no. 20/118, neg plasma), were obtained from NIBSC, South Mimms, UK. Sera and plasma were used for subsequent virus-specific mFIT and mVNT assays. Previously published purified recombinant henipavirus-specific mAbs (m102.4 [43], m5B3 [47]), RSV-F specific (mAb16, mAb18, mAb19, mAb20 [40], 101F [38], AM14 [36], 4D7 [37], motavizumab [39], MPE8 [35]; Table S1) mAbs, the SARS-CoV-specific mAb S230 [54] (Absolute Antibody) and a full-length recombinant ACE2-Fc were also used in virus-specific mFITs (all) and mVNTs (NiV, SARS-CoV and SARS-CoV-2).

### Cell–cell fusion assay

HEK293T Lenti rLuc-GFP 1–7 (effector cells) and HEK293T Lenti rLuc-GFP 8–11 (target cells) were seeded separately at 7.5×10^5^ per well in a six-well dish in 3 ml of PRF-DMEM-10% and incubated overnight at 37 °C, 5 % CO_2_. Transfection mixes were set up in 200 µl Opti-MEM (Gibco) with the *Trans*IT-X2 Dynamic Delivery System as per the manufacturer’s recommendations (Mirus). Viral glycoproteins (vGP; hRSV-F, bRSV-F, NiV-F+NiV G, SARS-CoV S or SARS-CoV-2 S) were transfected into effector cells and the corresponding receptor (hACE2 for SARS-CoV S and SARS-CoV-2 S) into target cells. For NiV and RSV, target cells were not transfected with receptor as HEK293T cells are already fusion competent. A mock-transfected (pcDNA3.1 empty plasmid, - vGP) and positive transfection control (250 ng rLuc-GFP 8–11 plasmid) was also set up. Transfections were incubated at 37 °C, 5 % CO_2_ for 24–48 h. Cells were then carefully washed with 2 ml fresh PRF-DMEM-10 % and harvested, and similarly transfected wells were pooled and diluted to 2×10^5^ ml^−1^. Diluted effector cells were added in 100 µl volume (2×10^4^ cell/well) to each well of a white-bottomed, sterile 96-well plate (Corning). Effector cells were then co-cultured with 100 µl of diluted target cells (2×10^4^ cell/well) for 18–24 h at 37 °C, 5 % CO_2_, after which GFP-positive syncytia and *Renilla* luciferase were quantified (see Methods: Luciferase assays and IncuCyte). Negative controls (effector cells only, target cells only) and positive transfection controls (HEK293T Lenti rLuc-GFP 1–7 cells transfected with rLuc-GFP 8–11 plasmid) were always included.

### Micro-fusion inhibition test (mFIT)

Effector and target cells were set up as described in the cell–cell fusion assay above. For the mFIT, sera or antibodies were diluted to optimized dilutions in sterile 1.5 ml tubes using serum-free PRF-DMEM and plated at 25 µl/well in a white-bottomed, sterile 96-well plate (Corning), including no sera/antibody controls. The sera/antibody were incubated with 2×10^4^ effector cells in 50 µl at 37 °C, 5 % CO_2_ for 1 h, after which target cells were co-cultured to corresponding wells and incubated for 18–24 h (Fig. 1, appendix 1: Quick mFIT protocol). Of note, for mAbs or polyclonal sera with unknown neutralization properties preliminary assays with a broad range of dilutions were performed to establish an initial working dilution, prior to the addition of effector cells for use in our standard mFITs. For hRSV-F and bRSV-F, this dilution was determined to be 1 : 160, and for NiV-FG, a 1 : 5 dilution of sera was shown to be the most inhibitory (Fig. S4c).

### IncuCyte

To quantify GFP expression, cells were plated in clear flat-bottomed 96-well plates (Nunc) and imaged every hour using the IncuCyte S3 live cell imaging system (Essen BioScience). Five fields of view were taken per well at 10× magnification, and GFP expression was determined using the total integrated intensity metric included in the IncuCyte S3 software (Essen BioScience). To analyse images generated on the IncyCyte S3, a collection of representative images is first taken to set fluorescence and cellular thresholds, which allows for the removal of background fluorescence, and selection of cell boundaries (‘objects’) by creating ‘masks’. Following this, the total integrated intensity metric can be accurately calculated by the software, which takes the total sum of objects’ fluorescent intensity in the image, expressed as green count units (GCU) µm^−2^.

### Generating lentiviral-based pseudoparticles

Lentiviral-based NiV pseudoparticles (NiVpp) were generated as described previously [48]. Briefly, HEK293T cells were transfected with NiV-G and NiV-F vGPs along with p8.91 (encoding for HIV-1 gag-pol) and CSFLW (lentivirus backbone expressing a firefly luciferase reporter gene). A ‘no-GP’ control was also set up using an empty plasmid. NiV-pp were titrated 10-fold on BHK-21 target cells and firefly luciferase activity was measured. To generate SARS-CoV pseudoparticles (SARS-CoV-pp) and SARS-CoV-2 pseudoparticles (SARS-CoV-2-pp), HEK293T cells were plated at 7.5×10^5^/well in six-well dishes and transfected the following day with 600 ng p8.91, 600 ng CSFLW and either 500 ng SARS-CoV S or 500 ng SARS-CoV-2 S with 10 µl PEI, 1 µg ml^−1^ (Sigma). No-GP controls were included using pcDNA3.1. The following day, media was replaced with 3 ml DMEM-10% and pooled harvests of supernatants containing SARS-CoV-pp and SARS-CoV-2-pp were collected at 48 and 72 h post-transfection, centrifuged at 1300 ***g*** for 10 min at 4 °C to remove cellular debris, aliquoted and stored at −80 °C. Pseudotype viruses were titrated 10-fold on HEK293T cells previously transfected with a hACE2 expression plasmid with their respective no-GP controls, plated 1 day prior to infection at 2×10^4^ in 100 µl DMEM-10 %. Firefly luciferase was quantified (see Methods: Luciferase assays). CSV files were exported onto a USB flash drive for subsequent analysis.

### Micro-virus neutralization test (mVNT)

Sera from NiV-vaccinated pigs were diluted in serum-free DMEM and 50 µl was added to a 96-well plate in triplicate (final dilution 1 : 20) and titrated fourfold or henipavirus-specific mAbs were added to a clear flat-bottomed 96-well plate in triplicate at 10, 1and 0.1 µg ml^−1^. NiV-pp were added at a dilution equivalent to 10^5^ signal luciferase units in 50 µl and incubated with sera or mAb for 1 h at 37 °C, 5 % CO_2_. BHK-21 target cells were then added at a density of 2×10^4^ in 100 µl and incubated at 37 °C, 5 % CO_2_ for 72 h. For SARS-CoV-2 and SARS-CoV mVNTs, convalescent plasma was diluted (final dilution 1 : 40) in serum-free DMEM and 50 µl was added to a 96-well plate in triplicate and titrated twofold. The S230 mAb and recombinant ACE2-Fc were added in triplicate at 50, 25, 12.5 and 6.25 µg ml^−1^. SARS-CoV-2-pp or SARS-CoV-pp was added at a dilution equivalent to ~10^6^ signal luciferase units in 50 µl and incubated with sera or Ab for 1 h at 37 °C. Target HEK293T cells previously transfected with a hACE2 expression plasmid were then added at a density of 2×10^4^ in 100 µl and incubated at 37 °C, 5 % CO_2_ for 72 h. Firefly luciferase activity was then measured (see Methods: Luciferase assays). CSV files were exported onto a USB flash drive for subsequent analysis.

### Luciferase assays

To quantify *Renilla* luciferase expression in fusion assays and mFITs, media were replaced with 100 µl of phosphate-buffered saline (PBS) followed by 60 µl of diluted substrate, Coelenterazine-H, 1 µM (Promega) 1 : 400 with PBS. The plate was incubated in the dark for 2 min then read on the GloMax Multi^+^ Detection System (Promega) To quantify firefly luciferase in mVNTs, media were replaced with 100 µl 1 × reporter lysis buffer and incubated at room temperature for 2 h on an orbital shaker. Then 45 µl of lysate was transferred to a white-bottomed 96-well plate (Corning) and 45 µl luciferase assay substrate. The plate was incubated in the dark for 2 min and then read on a GloMax Multi+ Detection System (Promega) as above. CSV files were exported onto a USB flash drive for analysis.

### Data analysis and statistics

For each sera/antibody, luciferase values are expressed as a percentage of the mean luciferase activity seen in the no-sera/antibody control. Data were plotted and analysed using GraphPad Prism v8.2.1. A percentage <100 correlates with a reduction of luciferase production and therefore inhibition of fusion (mFIT) or particle entry (mVNT). Serum neutralization titres in mVNTs were calculated as the inverse of the dilution at which there is 50 or 90 % inhibition of luciferase values, IC_50_/IC_90_. Statistical analysis was performed in GraphPad Prism v8.2.1.

## Supplementary Data

Supplementary material 1
