# Supplementary material for: Micro-fusion inhibition tests: quantifying antibody neutralization of virus-mediated cell–cell fusion
Source: J Gen Virol. 2020 Oct 15;102(1):jgv001506. doi: 10.1099/jgv.0.001506 (PMC8116787; doi:10.1099/jgv.0.001506)
Supplement: Supplementary material 1 [file jgv-102-506-s001.pdf]

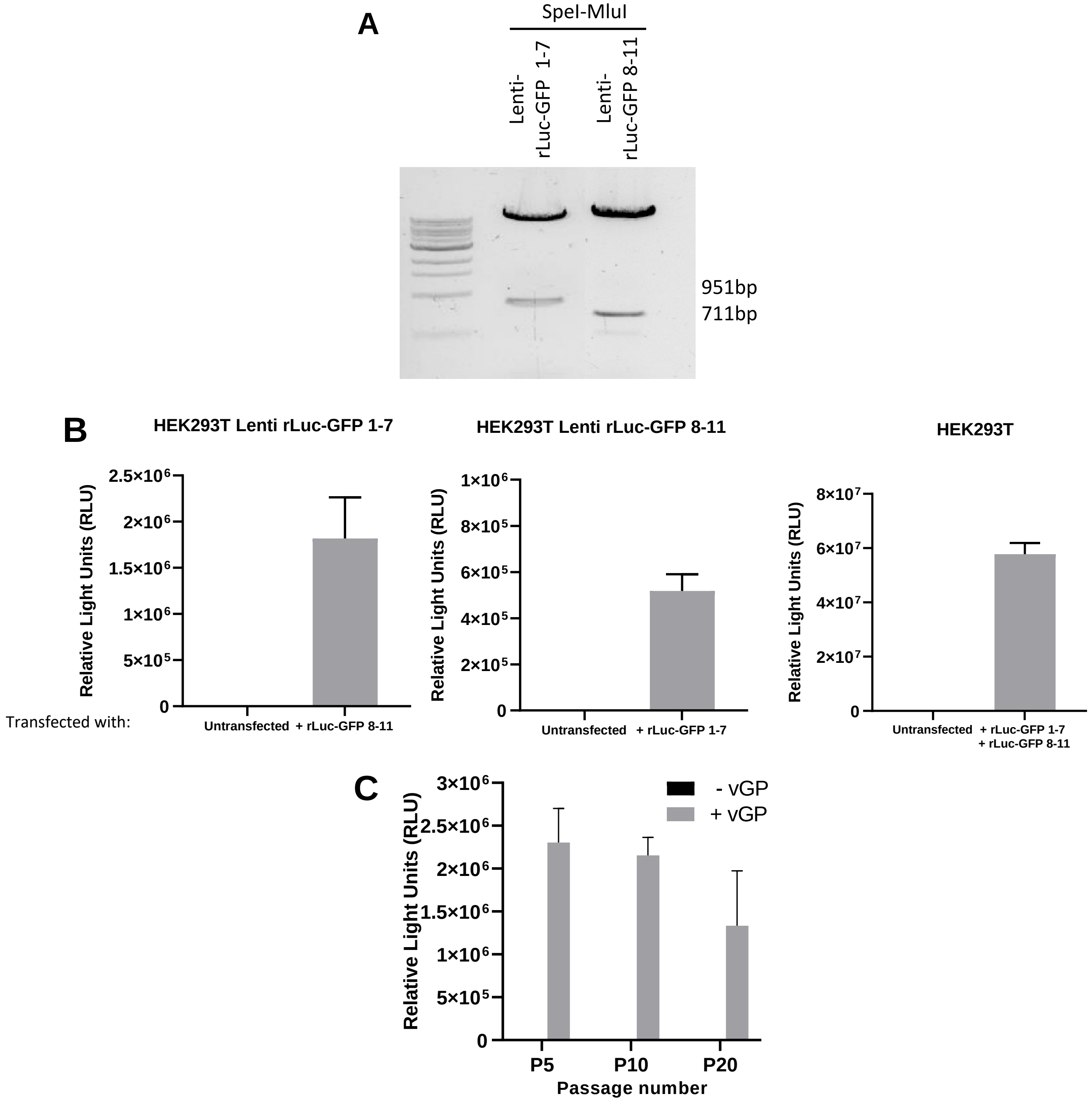

Supplementary Figure 1: Cloning of rLuc-GFP 1-7 and rLuc-GFP 8-11 into lentiviral vectors and testing the stability of these stable cell lines. To simplify our approach and minimize the manipulation of effector and target cell populations in the fusion assay, cell lines stably expressing either half of the split rLuc-GFP reporter were generated. **(A)** TBE DNA gel of rLuc-GFP 1-7 and rLuc-GFP 8-11 ORFs (gifted by Zene Matsuda, University of Tokyo) cloned into a lentiviral vector between *SpeI* and *MluI* restriction sites. Product sizes of 951bp (rLuc-GFP 1-7) and 711bp (rLuc-GFP 8-11) were seen following excision with the same enzymes. Transduction into HEK293T cells followed with puromycin selection. **(B)** The stable expression of Lenti rLuc-GFP 1-7 (**left**) or Lenti rLuc-GFP 8-11 (**middle**) in individually cloned HEK293T cells was tested by transfecting the corresponding half of the reporter (rLuc-GFP 8-11 and rLuc-GFP 1-7, respectively). This was compared to transient transfection of both rLuc-GFP 1-7 and rLuc-GFP 8-11 in HEK293T cells (**right**). Both the stable cell lines showed comparable levels of *Renilla* expression, while as expected untransfected cell lines did not express luciferase. **(C)** The expression stability of the introduced genetic elements was examined after 5, 10 and 20 passages of the resultant cell-lines. At each passage number, effector cells stably expressing Lenti rLuc-GFP 1-7 were either left untransfected (-vGP), or were transfected with 500ng of NiV-F and 500ng of NiV-G (+vGP). This cell population was co-cultured with target cells stably expressing Lenti rLuc-GFP 8-11. Luciferase values at each passage were comparable, demonstrating robust and continued expression of both components. Error bars represent mean + standard deviation.

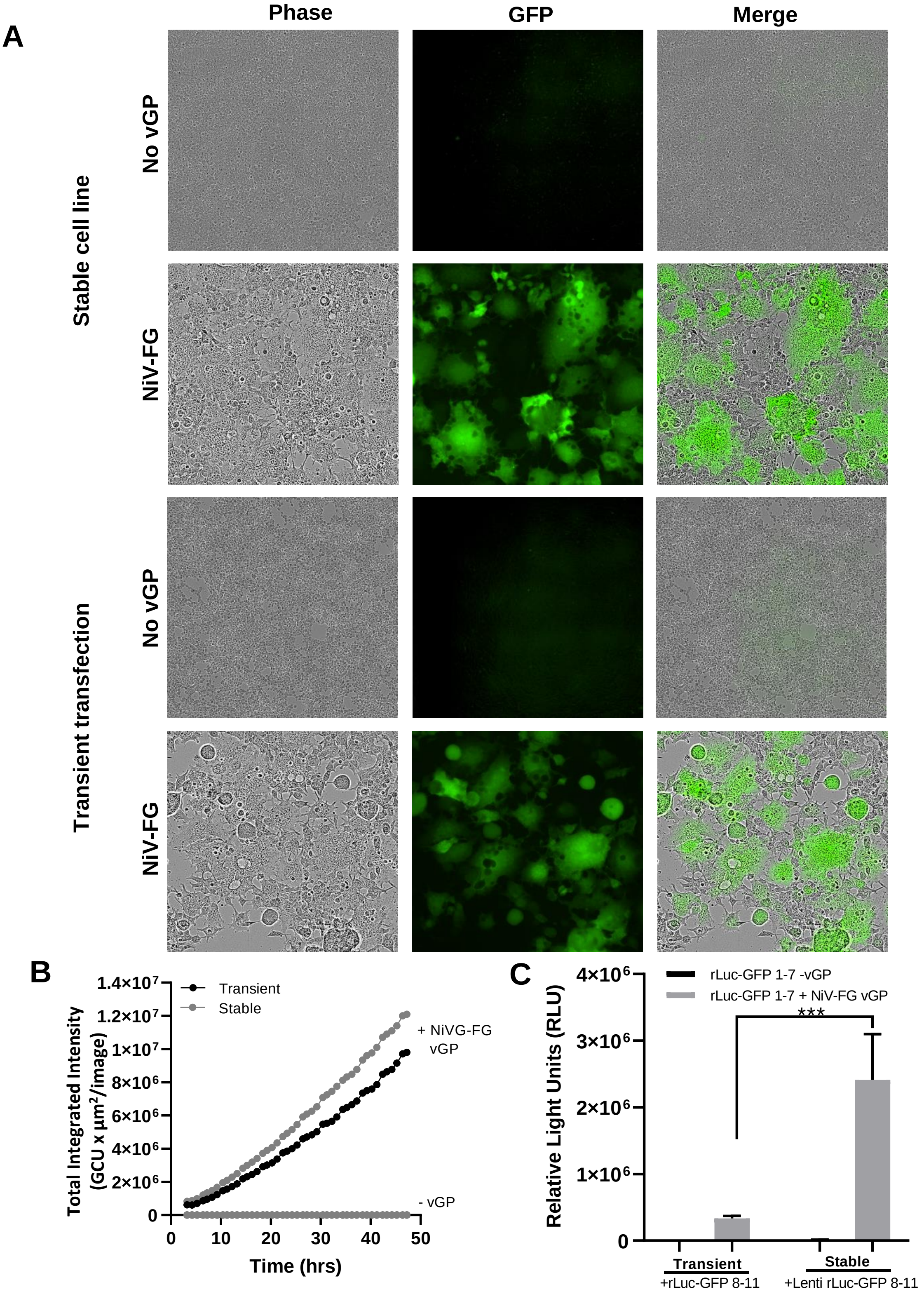

Supplementary Figure 2: Comparison of transient and stable expression of Lenti rLuc-GFP 1-7 and Lenti rLuc-GFP 8-11 in HEK293T cells in a NiV fusion assay. (A) Representative fluorescent GFP-positive syncytia in stably expressed (**top two rows**) or transiently transfected (**bottom two rows**) rLuc-GFP 1-7 (effectors) and rLuc-GFP 8-11 (targets) systems at 10x magnification. HEK293T effector cells were mock-transfected (No vGP, **top row for stable, third row for transient**) or transfected with 500ng each of NiV-FG vGP (**second panel for stable, bottom panel for transient**). Cells were then co-cultured with HEK293T target cells and GFP-positive syncytia were visualized together with **(B)** cell-cell fusion assay data showing GFP expression over time monitored using an IncuCyte live cell imaging system. The data plotted shows the total sum of syncytia fluorescent intensity in the image, calculated used the total integrated intensity metric and expressed as green count units (GCU) per μm^2. **(C)** *Renilla* luciferase readings in transiently transfected and stable cell-lines expressing the rLuc-GFP components in the presence or absence of NiV-FG vGP. Error bars represent mean + SD. Two-way ANOVA was used to compare luciferase expression in stable cell lines vs transient transfection, in the presence and absence of viral glycoprotein (*** $p<0.001$).

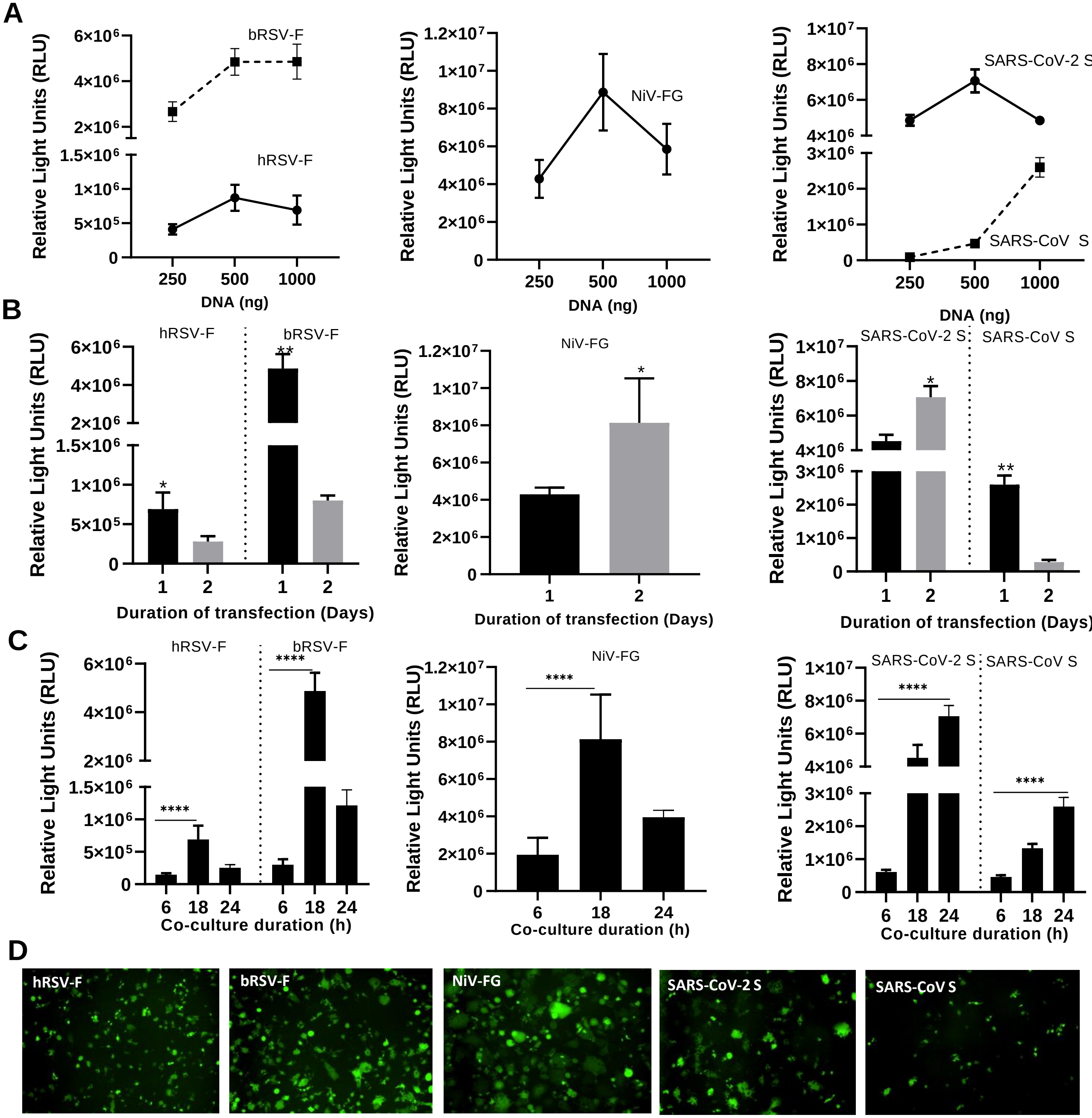

Supplementary Figure 3: Optimization of cell-cell fusion assays with viral glycoproteins. The conditions required for optimal fusion are rarely maintained between different vGPs. As such, various aspects of the cell-cell fusion assay must be optimized, which we show for human RSV, bovine RSV, Nipah virus, SARS-CoV and SARS-CoV-2, all using HEK293T cells stably expressing rLuc-GFP 1-7 and rLuc-GFP 8-11. **(A)** Transfected DNA mass for each vGP was optimized, with too much transfected DNA appearing inhibitory, perhaps due to vGP overexpression and cytotoxicity. **(B)** The length of time post-transfection (1 day or 2 days) prior to co-culture was also optimized. The kinetics of fusion can vary with NiV and SARS-CoV-2-effector cells being markedly more fusogenic 2 days post transfection, while for RSV and SARS-CoV, 1 day is better. This may be because NiV fusion, for example, requires two vGPs (the attachment (G) and fusion (F) proteins), whereas for RSV the F protein alone is sufficient for fusion. **(C)** For both NiV and RSV, the optimal duration of co-culture appeared to be 18 h, while for SARS-CoV and SARS-CoV-2 this was 24 h, with longer incubation times leading to excessive cell death. **(D)** Observed fluorescent GFP-positive syncytia in various fusion assays, at 10x magnification. Representative images are shown for each viral glycoprotein using the optimal fusion conditions calculated in A-C. Error bars represent mean $\pm$ SD. Paired t-test was used to compare luciferase expression 1-day vs 2-day post-transfection (** $p<0.005$, * $p<0.05$) and one-way ANOVA was used to test different co-culture duration times (**** $p<0.0001$, *** $p<0.001$).

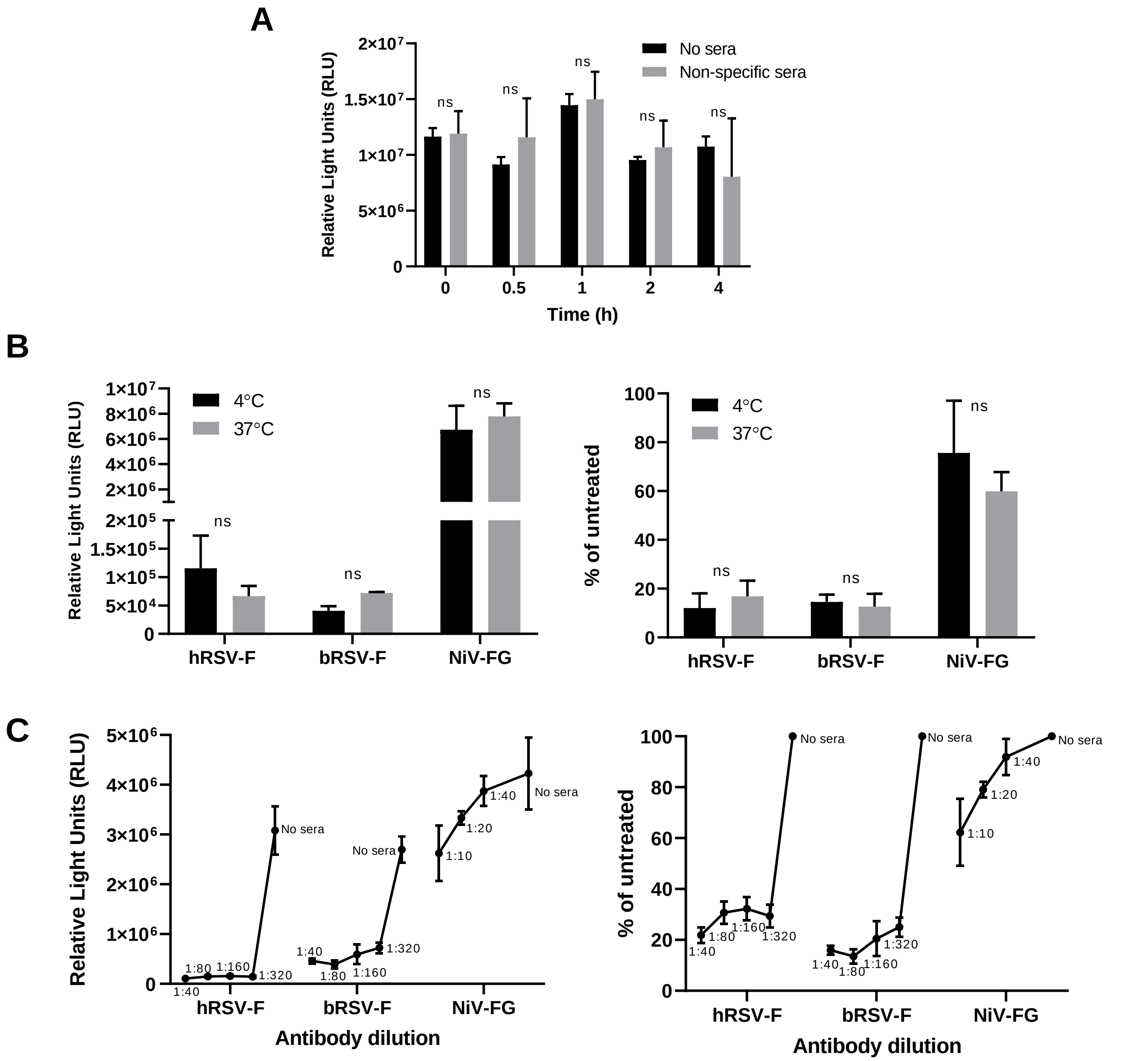

Supplementary Figure 4: Optimization of mFIT protocol using monoclonal antibodies and sera. Optimization of mFIT conditions, including **(A)** the duration of time for Ab incubation using non-specific sera and (**B**) the temperature at which effector cell incubation with antibodies against hRSV-F, bRSV-F or NiV-FG were performed. In **(A)** non-specific sera was used to establish the optimal co-culture duration in a NiV fusion assay, when compared to no sera controls. We noted that incubation for longer than 1 h was detrimental because of apparent non-specific effects of sera on the assay. In **(B)** we did not see marked differences in fusion inhibition when the effector cells were incubated at 4°C, so opted to incubate assays at 37°C (luciferase relative light units, RLU, **left**; RLU expressed as a percentage of untreated controls, **right**). **(C)** These conditions were then used to determine working dilutions for maximal inhibition of antibodies or sera, e.g. mAbs for hRSV-F and bRSV-F and polyclonal sera for NiV-FG, expressed as luciferase relative light units (RLU), **left** and expressed as a percentage of untreated controls (no sera = 100%), **right**. Error bars represent mean ± SD. Two-way ANOVA was used to test luciferase expression varying the time **(A)** or temperature **(B)** of co-culture conditions, ns = non-significant.

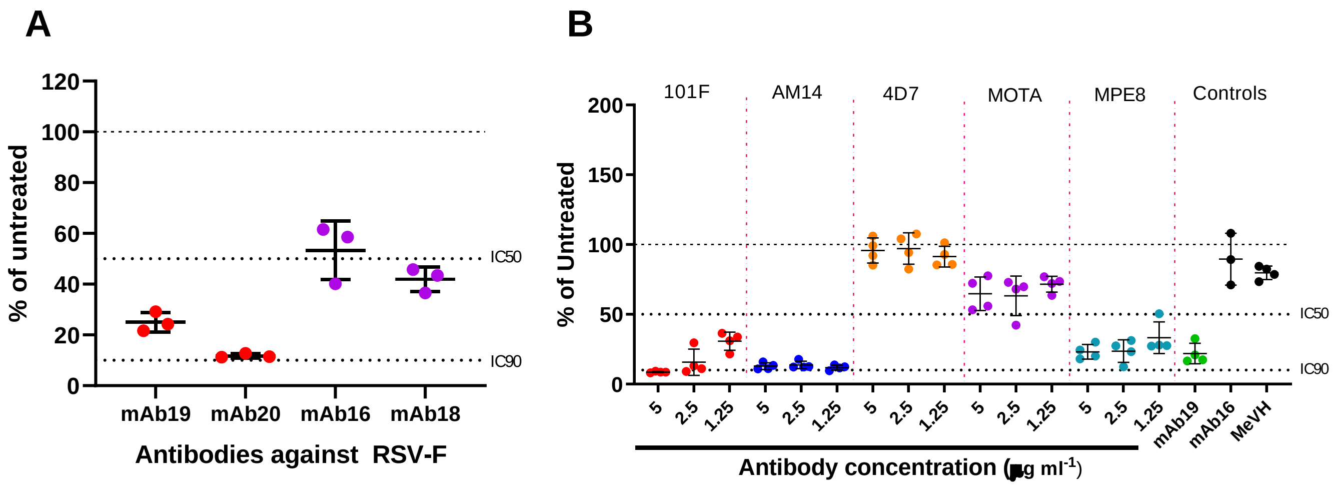

Supplementary Figure 5: Examining neutralization of fusion by monoclonal antibodies in bovine RSV mFITs. (A) Murine mAbs (1:160 working dilution) and **(B)** human mAbs (5µg ml^{-1}, 2.5µg ml^{-1}, 1.25µg ml^{-1}) were tested in bRSV-F mFITs. mAb 19 = positive control, mAb16 = specific negative control, MeVH = non-specific negative control. Data is expressed as a percentage of the average luciferase readings seen in no sera/negative controls with 50% or 90% inhibition (IC_{50} and IC_{90}) lines indicated. Error bars represent mean $\pm$ SD.

mAb	Antigenic site	Species	Pre-fusion/ post-fusion?	IgG class	Neutralizing	Fusion inhibitory	Ref
mAb16	**Site II**	Murine	Post	IgG1	Yes	No	40
mAb18	**Site II**	Murine	Post	IgG2a	Yes	No	40
mAb19	**Site IV**	Murine	Pre and post	IgG2a	Yes	Yes	40
mAb20	**Site IV**	Murine	Pre and post	IgG2a	Yes	Yes	40
4D7	**Site I**	Human	Post	IgG1	Yes	No	37
MOTA	**Site II**	Human	Pre and post	IgG1	Yes	Yes	39
MPE8	**Site III**	Human	Pre	IgG1	Yes	Yes	35
101F	**Site IV**	Human	Pre and post	IgG1	Yes	Yes	41, 38
AM14	**Site V**	Human	Pre	IgG1	Yes	Yes	36

Supplementary Table 1: Monoclonal antibody binding to RSV-F protein. Previously published data on the binding and neutralizing capability of various anti-RSV monoclonal antibodies. This includes information on known antigenic binding site of the pre- or post-fusion form of RSV-F, the species, the IgG subclass, and whether the monoclonal antibody is able to neutralize particle entry and/or fusion.

Days Post Immunization	0	7	14	21	28	35	42
**Adjuvant**							
**mFIT % inhibition**	0.0	0.0	11.7	15.4	11.3	0.0	28.5
**mVNT IC90**	<32	<32	<32	<32	<32	<32	<32
**NiVsG**							
**mFIT % inhibition**	13.2	3.7	53.6	45.8	89.4	92.9	79.8
**mVNT IC90**	<32	<32	89	2048	22528	21866	32768
**NiVmcsF**							
**mFIT % inhibition**	0.7	0.0	55.2	29.2	93.4	92.2	91.4
**mVNT IC90**	<32	<32	85	1024	6144	6144	3434
**Pearson's *r* = 0.66**							

Supplementary Table 2: Correlating NiV mFITs and pseudoparticle virus neutralization tests (mVNTs). Individual IC_{90} (mVNT) and mFIT percentage inhibition values from the immunogenicity studies performed in pigs, graphically presented in **Figure 3F**. The Pearson's correlation factor, r was calculated from the tabulated data correlating all available mFIT % inhibition and mVNT IC_{90} values.
